# The Association between Imaging Parameters of the Paraspinal Muscles, Spinal Degeneration, and Low Back Pain

**DOI:** 10.1155/2017/2562957

**Published:** 2017-03-20

**Authors:** Leonid Kalichman, Eli Carmeli, Ella Been

**Affiliations:** ^1^Physical Therapy Department, Recanati School for Community Health Professions, Faculty of Health Sciences, Ben-Gurion University of the Negev, Beer-Sheva, Israel; ^2^Department of Physical Therapy, Faculty of Social Welfare and Health Sciences, University of Haifa, Haifa, Israel; ^3^Department of Physical Therapy, Faculty of Health Professions, Ono Academic College, Kiryat Ono, Israel; ^4^Department of Anatomy and Anthropology, Sackler Faculty of Medicine, Tel Aviv University, Tel Aviv, Israel

## Abstract

This narrative review investigated imaging parameters of the paraspinal muscles and their association with spinal degenerative features and low back pain (LBP) found in the literature. Three principal signs of muscle degeneration were detected on imaging: decreased muscle size, decreased radiographic density, and increased fat deposits. Men have a higher density of paraspinal muscles than women, younger individuals have a higher density than older ones, and lean individuals have a higher density than those with an increased body mass index. Fatty infiltration appears to be a late stage of muscular degeneration and can be measured noninvasively by an MRI scan. Fatty infiltration in the lumbar multifidus is common in adults and is strongly associated with LBP, especially in women, independent of body composition. Fatty infiltration develops in areas where most degenerative changes are found. MR spectroscopy studies have corroborated that the lumbar multifidus in LBP subjects has a significantly higher fat content than asymptomatic controls. There is a strong need for establishing uniform methods of evaluating normal parameters and degenerative changes of the paraspinal muscles. Additional imaging studies are needed to improve the understanding of the association and causal relationships between LBP, spinal degeneration, and changes in the paraspinal muscles.

## 1. Introduction

Despite the very high prevalence of low back pain (LBP), its pathophysiology is poorly understood and there is a lack of an association between investigative findings and clinical symptoms [1]. LBP is defined as pain, muscle tension, or stiffness localized below the costal margin and above the inferior gluteal folds, with or without sciatica. Nonspecific LBP is defined as pain unattributed to a recognizable pathology (e.g., infection, tumor, osteoporosis, rheumatoid arthritis, fracture, and inflammation). Several studies have argued that the lack of an association between radiographic pathology and pain essentially stems from the multifactorial nature of pain (including various biological, psychological, and social factors). It is also possible that the poor association is due to factors that have not been evaluated by routine imaging, that is, degenerative changes in facet joints [2, 3], ligamentous damage [4], and changes (traumatic or degenerative) in the paraspinal muscles [5–8].

By the end of the last century, only scarce information detailing the role of the paraspinal muscles in the etiology of LBP was published [6, 9]. During the last decade, more and more studies have further explored the interaction between the paraspinal muscles, LBP, and spinal pathology. The abundance of these new studies entails a comprehensive approach to assist in summarizing the current data and knowledge.

Evaluating paraspinal muscles by computed tomography (CT), ultrasound (US), or magnetic resonance imaging (MRI) is not routine. One possible reason is the absence of simple and reliable measures of paraspinal muscle degeneration. Developing these measures may lead to an accumulation of empirical data and eventually to high-quality research studies focusing on the association between paraspinal muscle degeneration, spinal pathology, or LBP [10].

The aim of this narrative review was to determine, based on existing literature, normal and abnormal imaging parameters of paraspinal muscles (transversospinales, rotators, multifidus, and semispinalis) and the erector spinae (iliocostalis, longissimus, and spinalis) and their association with spinal degenerative features (disc degeneration, facet joint osteoarthritis, etc.) and LBP.

## 2. Methods

PubMed, Web of Science, CINAHL, Scopus, PEDro, and Google Scholar databases were explored, from inception until July 2016, using a predefined search strategy. The databases were searched for the following keywords: paraspinal muscles, multifidus, transversospinales, erector spinae, spine, spinal degeneration, and low back pain.

*Criteria for Inclusion.* Researches describing normal and abnormal presentation of paraspinal muscles and their association with spinal degenerative changes and LBP were included. Trials of any design and methodological quality were included. No language restrictions were imposed. The reference lists of all articles retrieved in full were also searched. The search results were pooled and duplicates removed. The titles and abstracts of all articles were reviewed. Full texts of potentially relevant papers were read and their reference lists searched for additional relevant articles. After excluding all irrelevant papers, a total of 67 publications were included in the review.

## 3. Results

### 3.1. Imaging Techniques Used in Studying Paraspinal Muscles

Morphological studies of paraspinal muscles are obtained by CT, MRI, and US imaging. Moreover, several imaging techniques have been used to study the fatty degeneration of the lumbar multifidus, including magnetic resonance (MR) spectroscopy, chemical shift MRI, and multiecho MRI [6, 11–13].

#### 3.1.1. CT

CT scans provide noninvasive and reproducible information related to muscle density, a cross-sectional surface area (CSA), and other muscle characteristics such as fatty infiltration [5]. Macroscopically, there are two foremost signs of muscle degeneration easily detected on CT images: a decrease in the size of the muscles and an increase in the amount of fatty deposits. In addition, muscle density can be measured by CT using the Hounsfield Unit (HU) [14]. Both Kalichman et al. [10] and Keller et al. [15] found that the reliability of the CT scan for measuring a CSA and the density of the back muscles in patients with chronic LBP is acceptable.

#### 3.1.2. MRI

An MRI scan provides noninvasive information as to muscle CSA and fatty infiltration and is generally undertaken when a tumor, infection, an insufficiency fracture, or disc protrusion is suspected. Convincing reliability was found when muscle-related MRI variables were evaluated. When muscles were graded, the interobserver agreement was fair to moderate, whereas intraobserver agreement was almost perfect [16, 17].

#### 3.1.3. MR Spectroscopy and Multiecho MRI

Schilling et al. [18] found that the spectroscopic results of the fat-water ratio correlated well with the histologic findings of muscle biopsies performed in two patients. The authors concluded that MR spectroscopy is a constructive method to detect metabolic changes in lumbar back musculature. Recently, Fischer et al. [11] proposed a novel approach by utilizing a multiecho MRI for quantification of lumbar multifidus fat content, concurring with the fat values derived by MR spectroscopy.

#### 3.1.4. US

Measurements of muscle size using US imaging have produced an accurate assessment of muscle wasting in various muscles [19]. The lumbar multifidus muscle, of particular interest, has been studied in normal subjects and in patients suffering from LBP. Performing an US to assess the multifidus size is a repeatable and reliable imaging technique in the hands of a trained assessor [9, 20] and, thus, can be used to compare different populations [21]. The validity of these measurements has been demonstrated [22].

Recently, Cuellar et al.'s systematic review [23] demonstrated that muscular measurements of older adults can be performed with moderate to substantial reliability using various imaging modalities (CT, MRI, and US). In summary, various imaging techniques (CT, MRI, and US) are reliable and useful tools for measuring CSA, density, and fatty infiltration of paraspinal muscles [6, 11–23].

### 3.2. Measurements of the Paraspinal Muscles

#### 3.2.1. CSA

A CSA can be measured either by CT, MRI, or US. The CSA is measured by total CSA, the atrophy ratio (functional CSA to total CSA), CSA asymmetry (as a percentage), fat CSA to a total CSA ratio, and the side-to-side difference in atrophy ratio [24].

Levels of a CSA evaluation vary significantly between studies. Some investigations refer to a single vertebral level, while others report a cross section up to 11 levels between L1 and S1. Within a single level, the reported CSA can be measured at the level of the center of the intervertebral disc, at the middle of the lamina, at the superior/inferior endplate, or at the center of the vertebral body [24–28].

Vast variability is also present in the orientation of the “slice.” Some authors adjusted the image parallel to the superior endplate/inferior endplate of the vertebral body; others used the CT reformatted image, perpendicular to the muscle mass/muscle fiber orientation [24–28]. Due to the above reasons, it is difficult to determine normal CSA values adjusted for age and gender in a healthy population and in individuals with LBP (Table 1).

#### 3.2.2. Muscle Density

**Figure 1:**
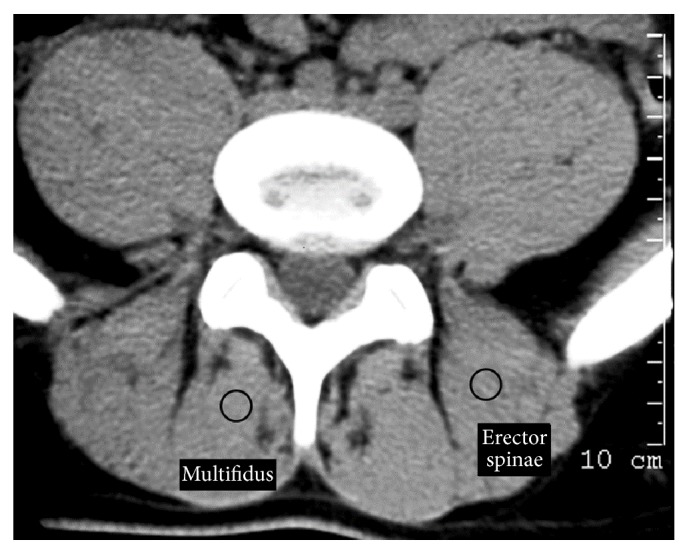
An example of a paraspinal muscle density evaluation using a 6 mm circle in the center of the most preserved muscle mass positioned on the noncontrast axial lumbar spine CT (L5-S1 spinal level) of a 34-year-old male subject.

Muscle density is usually measured by CT. Most studies use HU to evaluate the density of the muscle fibers. When evaluating muscle density, the same problem occurs as when measuring the CSA, that is, including a large variability of spinal levels and different orientations of the “slice.” Moreover, some studies presented data as to the muscle density of the entire muscle, while others furnished data for a small part of the muscle (6 or 10 mm circle) in the center of the most preserved muscle mass (Figure 1) [5, 10, 15, 28, 29] (Table 2); therefore, it is difficult to determine normal and abnormal parameters of muscle density.

#### 3.2.3. Fatty Infiltration

The aging process causes skeletal muscle mass to decrease and be replaced by noncontractile connective tissue. This age-related muscle atrophy [30], known as sarcopenia, seems to be due to a reduction in both number and size of muscle fibers, mainly the fast twitch muscle fibers, Type IIX, and is to some extent caused by a slowly progressive neurogenic process. Moreover, the age-related alteration in the differentiation potency of myogenic adult stem cells, known as satellite cells, which differentiate to fat cells instead of myocells, resulted in the accumulation of intermuscular fat/adipose tissue [31].

Studies have shown that fatty infiltration appears at a late stage of muscular degeneration and is associated with stroke, spinal cord injury, diabetes, and COPD. MRI, MR spectroscopy, or US can measure fatty infiltration in a noninvasive manner. The methods used to assess fatty infiltration can be classified as either a visual semiquantitative assessment or quantitative measurements.

#### 3.2.4. Visual Semiquantitative Assessment of Fatty Infiltration

**Figure 2:**
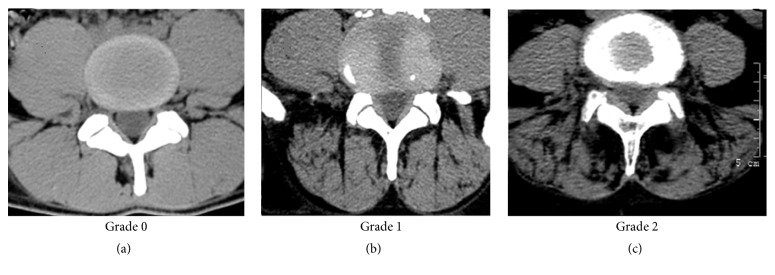
An example of different fatty infiltration grades in lumbar paraspinal muscles observed on a lumbar spine CT, imaged with a 64-slice CT scanner (Philips Medical, Brilliance Power 64). (a) A 23-year-old male; (b) a 61-year-old male; (c) a 72-year-old female.

Solgaard Sorensen et al. [17] visually graded fatty infiltration using the standard criteria in adults: 0 (no fat), 1 (slight infiltration), and 2 (severe infiltration) if present at one or more lumbar levels. Kalichman et al. [10] defined the assessment as more quantitative: Grade 1: a normal muscle condition, fatty infiltration up to 10% of the muscle's CSA; Grade 2: moderate muscle degeneration, 10–50% of fatty infiltration; Grade 3: severe muscle degeneration, >50% of fatty infiltration (Figure 2). The authors found high intrarater and interrater reliability. Kjaer et al. [16] employing the same method, found that the intraobserver and interobserver reliabilities for adults were satisfactory; however, for adolescents, the visual assessment of fatty infiltration was unsatisfactory and should be interpreted with caution. It may, therefore, be difficult to establish the extent of fatty infiltration in muscles by mere visual inspection.

By adapting the more detailed 5-grade Goutallier classification system [32] for grading lumbar multifidus fatty degeneration found on an MRI, it offers two distinct advantages over the mild, moderate, and severe classification identified on a CT. Firstly, this method is semiquantitative and provides a numerical scale for fat content and secondly, an MRI is favored over a CT in a diagnostic workup of acute and chronic LBP.

#### 3.2.5. Quantitative Methods

Recently, a number of studies have quantified the fatty infiltration of the paraspinal muscles. Each study employed slightly different measurements in defining fatty infiltration. Fortin et al.'s [33] MRI study measured fatty infiltration in two different ways: (1) the ratio of fat CSA to total CSA as an indicator of muscle composition (or fatty infiltration) and (2) signal intensity as an indicator of fatty infiltration. In another MRI study, Hebert et al. [34] also used signal intensity to separate muscle from fat. Their results are presented as percentages of the fat CSA from the total muscle CSA. Niemeläinen et al. [35] used the ratio of fat CSA to total CSA as an indicator of muscle composition similar to Fortin et al. [33]. Chan et al. [27] referred to fat CSA (cm^2^) as an indicator of fatty infiltration.

Utilizing so many different methods for assessing and measuring fatty infiltration renders it impossible to compare values between the various studies. Future research should target applicable and clinically relevant redefinitions for fatty infiltration by taking into account age and sex differences.

### 3.3. Normal Imaging Parameters of Paraspinal Muscles

Data regarding the CSA of paraspinal muscles in healthy individuals and those with LBP is presented in Table 1. Despite the vast variability in measurement techniques, many researchers concur that the CSA of the multifidus in healthy subjects is larger in the lower lumbar segments and smaller in the upper lumbar segments [19, 24, 33, 35, 36]. On the other hand, a study revealed that the CSA of the erector spinae is smaller in the lower lumbar segments and larger in the upper lumbar segments [35]. It has also been suggested that paraspinal muscle asymmetry >10% was commonly found in men without a history of LBP [35].

Data on radiographic density of the paraspinal muscles in healthy individuals and those with LBP is presented in Table 2. Although comparing the results of the different studies is difficult due to methodological differences, most studies agree that the paraspinal muscle density is higher in men and that it decreases with age and an increased BMI [5, 28, 29].

Data on fatty infiltration in paraspinal muscles in healthy individuals and those with LBP is presented in Table 3. The amount of intramuscular fat significantly increased in the lower lumbar segments for the multifidus and the erector spinae muscles compared with the upper lumbar segments. Men show lower fatty infiltration in the paraspinal muscles than women. It is important to note that paraspinal muscle asymmetry >10% was commonly found in men without a history of LBP [29, 35].

We believe that additional studies are needed to establish the normal parameters of the paraspinal muscle density for males and females and for different age groups which would enable identification of a pathological deviation in parameters of muscle density and the development of prevention and treatment strategies for spinal degeneration conditions. The aforementioned data provided reference ranges for an objective assessment of lumbar paraspinal muscles. If US is to be adopted for use in routine musculoskeletal medicine/physiotherapy practice, it is important that the methodology for obtaining and measuring images will be standardized to ensure that the technique is robust and reliable. Secondly, shape varied considerably amongst normal subjects suggesting that it may be futile to refer to a typical shape. An assessment of the paraspinal muscle' size can be achieved by comparing the reported 95% reference ranges. Separate data are needed for each gender and vertebral level. Changes in the quality of muscle tissue with age require further investigation.

### 3.4. An Association between Degeneration of Paraspinal Muscles and Personal Characteristics

#### 3.4.1. Age

Surprisingly, only a few publications have described age-related changes in the paraspinal muscles. Most demonstrated a decrease in the CT-evaluated muscle density [5, 10, 37, 38] and the CSA of the multifidus and erector spinae [39–41]. On the other hand, US studies [19, 42] showed no association between the size of the multifidus and age. There were, however, in some cases, qualitative differences observed in terms of greater echogenicity with increasing age [19]. The discrepancy between US and CT/MRI findings can perhaps be explained by the CSA evaluation method. Usually, in CT/MRI CSA measurements, the fatty infiltration is not considered part of the muscles, but in US evaluations, the entire muscle area (including fat) is measured.

In conclusion, CSA and the quality of paraspinal muscles decrease with age, most probably as an expression of age-related sarcopenia [43] in the paraspinal muscles. Age should be used as a covariate in studies evaluating the association between the paraspinal muscles, spinal degeneration, or LBP.

#### 3.4.2. Sex and Body Composition

Paraspinal muscle CSA and density are higher in men than in women [5, 10, 37]. In a US study by Stokes et al. [19], males had a significantly greater multifidus CSA but when normalized for body mass, no significant gender difference emerged. This should be confirmed by additional studies.

Kalichman et al. [5] found low but a statistically significant negative correlation between paraspinal muscle density and body mass index (BMI) (*r* = −0.193, *p* = 0.009 in transversospinalis and *r* = −0.251, *p* = 0.001 in erector spinae). Interestingly, in males the association between the paraspinal muscle density and BMI was insignificant; however, in females, it was moderate (*r* = −0.345, *p* = 0.002 in transversospinalis, and *r* = −0.390, *p* < 0.001 in erector spinae) (unpublished data). A similar negative association was found between the CT-evaluated muscle density measured in the mid-thigh and BMI [44].

On the other hand, no association has been found between fat deposits in the back muscles when evaluated by MRI and weight [16, 40]. Body fat in obese individuals is naturally deposited in the muscles throughout the back musculature and does not settle in the last two lumbar levels where most spinal abnormalities generally tend to cluster. The fact that fatty infiltration is mainly found in these two “troubled areas” tends to indicate that it is the LBP that initiates the muscle changes and not vice versa.

Other personal factors such as smoking, diabetes mellitus Type II, cardiovascular disease, and activity level have the potential of influencing CSA and fatty infiltration of the paraspinal muscles. Additional studies are needed to evaluate these influences.

### 3.5. An Association between the Degeneration of Paraspinal Muscles and Other Spinal Degeneration Features

#### 3.5.1. Lumbar Disc Herniation

Lumbar disc herniation is one of the most common diseases of the lumbar spine. The compression by a protruding disc on the dorsal and/or ventral rami of the nerve roots causes LBP, leg pain (sciatica), muscle spasms, and trunk movement restriction [45]. In patients with lumbar disc herniation, dysfunction of the back muscles is common. Multifidus atrophy has been reported in patients with LBP [5] and lumbar disc herniation [12]. Kim et al. [46] showed a decrease in multifidus CSA on the lesion side of patients with unilateral sciatica caused by lumbar disc herniation and suggested that the decrease in the CSA was related to the duration of the neural compression. This finding can be explained by the unilateral and segmental innervation pattern of the lumbar multifidus muscle [27].

Yoshihara et al. [47] studied the multifidus muscle in patients with an L4-L5 lumbar disc herniation. Significant decreases in the size of Type I (slow-twitch oxidative) and Type II (Type IIX/MHC-2X fibers, “fast twitch glycolytic”) (FG), together with structural changes, were demonstrated on the affected side of the L5 muscle band, where neural changes are expected to occur. These results suggest that nerve root impairment may lead to atrophy of Type I and Type II/MHC-2X fibers, with structural changes in the multifidus only at the involved level.

A histological study [48] exhibited a variety of neurogenic and myogenic changes in both diseased and normal sides of the multifidus after lumbar disc herniation. Both Type I and Type II fibers on the diseased side were significantly smaller than those on the normal side. Pathological findings (fiber-type grouping, small angulated fibers, group atrophy, moth-eaten appearance, intermyofibrillar network irregularity on nicotinamide-adenine dinucleotide tetrazolium reductase (NADH-TR) stained biopsy specimens, and internal nuclei) on the diseased side were more severe than those on the normal side. Type I fibers on the diseased side were significantly smaller when the symptoms were central low back pain.

An MRI study of 72 LBP patients [49] showed that a high percentage of fat in the multifidus was significantly associated with an increased risk of Modic change. Substantial fat replacement of the erector spinae was significantly associated with reduced intervertebral disc height and an increased risk of Modic change.

On the other hand, Kader et al. [1] found a significant correlation between multifidus muscle atrophy and leg pain in a retrospective MRI study of 78 patients (aged 17–72) with LBP. However, the relationships between muscle atrophy and radiculopathy symptoms, nerve root compression, herniated nucleus pulposus, and a number of degenerated discs was found statistically nonsignificant. Muscle degeneration was usually bilateral and multilevel, even in patients with a single nerve root irritation. In a German study [18], 10 patients with lumbar disc herniation and 16 healthy volunteers underwent proton MR spectroscopy (H-MRS). Patients with lumbar disc herniation demonstrated a significantly increased fat-water ratio of 0.19 compared to 0.09 in the control group (*p* value = 0.003).

In an experimental porcine model study of muscle changes after a lumbar spinal injury, Hodges et al. [52] found disc and nerve lesions. These data answered the query as to why the multifidus CSA quickly diminishes after a lumbar injury. Such changes may be due to disuse following reflexed inhibitory mechanisms.

In summary, there is a significant body of evidence explaining segment-specific degenerative changes in the lumbar multifidus after disc herniation, that is, a decrease in the multifidus CSA (especially on the lesion side), a decrease of muscle density (perhaps because of an increased fat-water ratio), and a decrease in the size of Type I and Type II/MHC-2X fibers and interstitial fibrosis.

#### 3.5.2. Facet Joint Osteoarthritis

Kalichman et al. [5] evaluated the association between the density of paraspinal muscles and facet joint osteoarthritis. When data were separately analyzed for each spinal level, the results showed a significant association between L4 multifidus/erector spinae density and facet joint osteoarthritis at L4-L5. Higher grades of facet joint osteoarthritis were found associated with a lower density of paraspinal muscles. In another study, Kalichman et al. [10] showed that after adjustment for age, sex, and BMI, facet joint osteoarthritis was significantly associated with low density and higher grades of fatty infiltration in the multifidus and erector spinae.

#### 3.5.3. Spondylolysis and Spondylolisthesis

Spondylolysis is an anatomical defect or fracture in the vertebral pars interarticular and is most commonly observed in the lowest lumbar vertebrae. Spondylolisthesis refers to the displacement of a vertebral body on the one below it and has several etiologies, the most common being spondylolysis (isthmic spondylolisthesis) and spondylotic (associated with the degeneration of the posterior facet joints and/or intervertebral disc) degeneration (degenerative spondylolisthesis). In Kalichman et al.'s [5] CT study, a significant association was found between the lower density of the multifidus muscle at level L4 and spondylolisthesis at the same level.

A recent Chinese study [53] evaluated the MRIs of 149 middle-aged degenerative spondylolisthesis patients and the same total of age- and sex-matched controls. The multifidus muscle atrophy ratio of the patients tended to be significantly lower than those in the control group, whereas the signal intensity ratio of the paraspinal muscles and the erector spinae muscle atrophy ratio were significantly higher than in the control group. Using a multivariate logistic regression analysis, it was confirmed that the erector spinae atrophy ratio and the signal intensity ratio of multifidus were independent predisposing factors to lumbar spondylolisthesis (OR > 1, *p* < 0.05) while the multifidus atrophy ratio was independent of protective factors (OR < 1, *p* < 0.05).

Another MRI study from India [41] assessed the CSA of the lumbar paraspinal muscles in 120 adults with isthmic spondylolisthesis. Compared with normal controls, the mean CSA value for the erector spinae was significantly higher in the study cohort (*p* = 0.002), whereas the CSA for the multifidus muscle was significantly lower (*p* = 0.009).

Additional studies are needed, but we can cautiously conclude that patients with spondylolisthesis (isthmic or degenerative) suffer from segmental atrophy of the multifidus muscle. The presence of erector spinae hypertrophy could be a mechanism to compensate for this instability.

#### 3.5.4. Lumbar Spinal Stenosis

Lumbar spinal stenosis generally refers to a compilation of symptoms associated with size reduction of the lumbar spinal canal or intervertebral foramina. This anatomical finding is essential for diagnosis, but the degree of stenosis is poorly correlated with symptom severity and functional impairment [54]. Chan et al. [27] studied 66 stenosis patients with no mechanical back pain or segmental instability. The authors found that the male stenosis patients exhibited a larger psoas relative CSA than the females, whereas the older patients exhibited a smaller psoas relative CSA and a higher multifidus fatty infiltration than the younger patients. Spinal stenosis patients in the high functional performance group exhibited a significantly larger psoas relative CSA and lower multifidus fatty infiltration. Patients with an increased multifidus fatty infiltration exhibited a significantly poorer functional performance suggesting that multifidus fatty infiltration was more representative of a neural injury than the degree of lumbar stenosis. It also suggests that multifidus fatty infiltration can be used as a prognostic factor of functional performance in spinal stenosis patients instead of the severity of spinal canal stenosis.

#### 3.5.5. Lumbosacral Ligamentous Damage

Jinkins's [55] MRI study compared the findings of 100 patients with LBP to those of 10 young asymptomatic volunteers. Associated paraspinal muscle (e.g., interspinales and multifidus muscles) degeneration was observed in a minority of overall cases (7%) but was only seen in cases demonstrating an interspinous ligament degeneration/rupture (10%).

### 3.6. The Association between Paraspinal Muscle Degeneration and Nonspecific LBP

There is a growing body of evidence showing an association between paraspinal muscle degeneration and LBP. Early studies report atrophy and other abnormalities of the paraspinal muscles in 20% to 60% of individuals with chronic LBP [56, 57]. In these patients, studies have consistently shown a decrease in paraspinal muscle CSA [1, 50], especially in the multifidus [9], attaining 10% compared to healthy individuals [52]. MRI [1] and CT [50] studies have observed multifidus atrophy in patients with chronic LBP and shown that atrophy was selective for multifidus. Neither the psoas nor erector spinae muscle masses were significantly smaller compared with the matched controls. On the other hand, Barker et al. [58] found a significant difference in the CSA of the multifidus and psoas between the symptomatic and asymptomatic sides of LBP patients.

Numerous studies have challenged the belief that an association exists between paraspinal muscle degeneration and LBP [59]. Fortin et al. [24] in a longitudinal study reported that variations in paraspinal muscle morphology shown on MRI have a limited, if not uncertain role in the short- and long-term predictions of LBP in men. D'Hooge et al. [25] found no difference in multifidus CSA between individuals with LBP and controls. In a recent comprehensive review, Cuellar et al. [23] reported no association between muscle size and LBP in older adults.

In a recent MRI study [49] of 72 adults, multifidus or erector spinae CSA was found unassociated with LBP or disability. A high percentage of fat in the multifidus (but not in the erector spinae) was found associated with an increased risk of high-intensity pain/disability. It is possible that LBP leads to altered neuromuscular functioning, which in turn causes changes in muscle histology, seen as atrophy [16]. However, the CSA of the muscle may not decrease due to the fatty infiltration in the muscle bundle.

The results of Kjaer et al.'s [16] MRI study of a large population sample presented convincing evidence that fatty infiltration in the lumbar multifidus is strongly associated with LBP in adults. This association was not affected by BMI, type of work, or level of physical activity during leisure time. However, the associations seem to be more pronounced in women. It is essential to investigate, in prospective studies, the causal relationships between fatty infiltration and LBP. It would also be constructive to evaluate whether the fatty infiltration in the lumbar multifidus is reversible and if so, whether this reversibility coincides with the improvement of symptoms.

Chronic LBP was also found to be associated with reductions in muscle fiber density [7]. In healthy individuals, the paraspinal muscles contain a high proportion of slow-twitch, low tonic, fatigue-resistant fibers (Type I) and are relatively larger in diameter than Type II/MHC-2X fibers, reflecting their role in maintaining posture and joint stability.

The percentage of Type I fibers is higher in females, leading to a better adaptation to aerobic exertion compared to males. Abnormalities seen in paraspinal muscles in patients with chronic LBP include Type II fiber atrophy, conversion of Type I to Type II fibers, and an increased number of nonspecific abnormalities. The extent of muscle changes is not necessarily related to symptom duration [59, 60].

Pathological changes in the internal fiber structure were more frequently encountered in older patients and were independent of symptom duration. The results suggest that over the long-term, fiber-type transformations rather than alterations in fiber size are the predominant changes found in the muscles of chronic LBP patients. The direction of change supports the results of previous studies that have demonstrated corresponding differences in the fatigability of the muscles [61, 62].

In patients with acute LBP, Hides et al. [51] found a marked wasting of the multifidus on the symptomatic side isolated to one vertebral level. The authors proposed that the wasting was unlikely due to disuse atrophy because of the rapidity of onset and localized distribution. Furthermore, greater multifidus atrophy was present in subjects with LBP and radiculopathy compared to those with only LBP [63].

### 3.7. Is It Possible to Reverse Degenerative Changes in Paraspinal Muscles?

After a review of the literature on the degeneration of paraspinal muscles, one question is always raised: Is it possible to reverse paraspinal muscle degeneration? A few papers have addressed this question. Kim et al. [64] evaluated the efficacy of spinal stabilizing exercises in reducing atrophy of the multifidus and psoas major muscles, reducing the levels of pain and disability and increasing paraspinal muscle strength in patients with degenerative disc disease. After eight weeks of spinal stabilization exercises, the paraspinal muscle strength significantly increased. The CT-evaluated CSAs of the multifidus and the psoas major increased compared with the preexercise size.

Another pre-post designed study [65] evaluated the effect of a staged stabilization-training program on lumbar multifidus CSA (using US imaging) in elite Australian cricketers with LBP. The stabilization program involved a voluntary contraction of the multifidus, transversus abdominis, and pelvic floor muscles with real-time feedback from the US imaging, non-weight-bearing to weight-bearing positions, and movement training. The CSAs of the multifidus muscles at the L5 vertebral level increased for the 7 cricketers with LBP who had received stabilization training compared with the 14 cricketers without LBP who did not receive rehabilitation (*p* = 0.004). In addition, the amount of muscle asymmetry amongst those with LBP significantly decreased (*p* = 0.029) and were found comparable to the cricketers without LBP.

In a small randomized controlled trial (RCT) [66], the effect of exercise on back muscle CSA, density, and strength was evaluated in 24 patients (11 cases and 13 controls) sick-listed for subacute LBP. Patients in the exercise group followed a biweekly exercise protocol for 15 weeks. Control patients received the usual care. Muscle CSA and density were measured by CT before and after the intervention. An isokinetic test of back extensors was simultaneously conducted. Results showed a tendency to increased muscle CSA and density in patients in the exercise group, in addition to a significant decrease in muscle CSA at L4-L5 in the controls and a significant difference in change between groups in muscle CSA at L4-L5. Back extension strength increased in patients in the exercise group; however, the improvement was not significant compared to the controls.

Another RCT [67] was performed comparing muscle strength, CSA, and density of back muscles in 124 patients with chronic LBP, disc degeneration, and postlaminectomy syndrome, randomized to either lumbar fusion or cognitive intervention exercise groups. The cognitive intervention group were told that ordinary physical activity would not harm the disc with a recommendation to bend the back when exercising. This was reinforced by three daily physical exercise sessions for 3 weeks. After a one-year follow-up, the exercise group performed significantly better in muscle strength than the lumbar fusion group. The density at L3-L4 decreased in the lumbar fusion group but remained unchanged in the exercise group. The CSA was unchanged in both groups. Inferring from the aforementioned studies, we can cautiously state that an intensive exercise program may improve strength, density, and CSA of paraspinal muscles in subjects with LBP.

## 4. Conclusions

Three principal signs of muscle degeneration were detected on imaging: a decrease in the size of the muscle CSA, a decrease in radiographic density, and an increase in the amount of fat deposits. The results of this review demonstrate that men have a larger CSA and higher density of paraspinal muscles than women, younger individuals have a higher density than older ones, and individuals with less weight have a higher density of paraspinal muscles than those who are overweight.

Segment-specific degenerative changes in the lumbar multifidus and erector spinae after disc herniation are associated with the duration of neural compression. A level-specific association was found between facet joint osteoarthritis and density of the multifidus and erector spinae and between the density of the multifidus and spondylolisthesis.

Fatty infiltration and accumulation appear to be a late stage of muscular degeneration or age-related muscle changes and can be measured in a noninvasive manner by MRI. Fatty infiltration in the lumbar multifidus is common in adults and strongly associated with LBP, especially in women, appearing to be independent of body composition and developing in areas where most degenerative changes are found. MR spectroscopy confirmed that the lumbar multifidus in LBP subjects had a significantly higher fat content than in the asymptomatic controls.

There is a strong need for establishing uniform methods of evaluating degenerative changes of the paraspinal muscles. Additional studies are needed to improve the understanding of the association and causal relationships between LBP, spinal degeneration, and changes in the paraspinal muscle. Accurate identification of the origin of LBP can potentially provide a more rational approach to patient management.

## Figures and Tables

**Table 1:** Cross-sectional area of back muscles and association with LBP.

Research	Modality	Participants	Segments measured	Level of measurement	Position	Orientation of cross section	CSA multifidus (cm^2^)	CSA erector spinae (cm^2^)	Association with LBP
Danneels et al. [50]	CT	23 healthy volunteers	L3 L4 L4	Superior endplate Superior endplate Inferior end plate	Supine	Adjacent to the vertebral endplate	4.7 ± 1.4 6.3 ± 1.4 9.0 ± 1.5		A significant difference between the two groups, especially at the L4 inferior endplate. Healthy individuals have a larger CSA of the multifidus
		32 patients with LBP	L3 L4 L4	Superior endplate Superior endplate Inferior end plate			4.1 ± 1.0 5.7 ± 1.1 7.7 ± 1.4		

Hides et al. [51]	US	10 young male elite cricketers with LBP	L2 L3 L4 L5	Spinous process of the vertebra	*Prone* with flattened lumbar lordosis	Between the spinous process and the lamina	3.4 ± 1.4 5.1 ± 1.9 7.1 ± 2.7 7.4 ± 2.1		Multifidus muscle atrophy can exist in highly active, elite athletes with LBP. Specific retraining resulted in an improvement in multifidus CSA that was concomitant with pain decrease
		16 young male elite cricketers asymptomatic	L2 L3 L4 L5				2.8 ± 1.1 4.3 ± 1.5 6.5 ± 2.2 8.0 ± 1.7		

Stokes et al. [19]	US	68 females	L4 L5		*Prone* with flattened lumbar lordosis	Between the spinous process and the lamina	5.6 ± 1.3 6.7 ± 1.0		
		52 males	L4 L5				7.9 ± 1.9 8.9 ± 1.7		

Chan et al. [27]	US	12 asymptomatic men	L4	Vertebral lamina	Prone		6.16 ± 0.09		Smaller multifidus CSA in chronic LBP patients than that in controls at all postures
					Standing		7.16 ± 0.10		
		12 men with LBP	L4		Prone		5.37 ± 0.06		
					Standing		6.58 ± 0.20		

Fortin et al. [33]	MRI	33 patients diagnosed with posterolateral disc herniation at L4-L5	L3-L4 L4-L5	The center of each intervertebral disc	Supine	Perpendicular to the muscle mass	6.5 ± 1.4 9.6 ± 2.1	20.0 ± 4.4 16.3 ± 4.1	There was no significant asymmetry of the multifidus at spinal level above, same level, or level below the disc herniation
			L5-S1 S1	The center of S1 vertebral body			11.7 ± 2.3 13.2 ± 2.7	10.2 ± 4.1 9.1 ± 4.0	

D'Hooge et al. [25]	MRI	13 individuals with recurrent nonspecific LBP, and 13 asymptomatic individuals	L3 L4 L4	Superior endplate Superior endplate Inferior end plate	Supine	Adjacent to the vertebral endplate	Normalized values to L4 superior endplate		No difference in CSA between individuals with LBP and controls

Niemeläinen et al. [35]	MRI	126 asymptomatic men	L3-L4 L4-L5 L5-S1	Not described in the manuscript	Supine	Not described in the manuscript	Rt: 7.3, Lt: 6.9 Rt: 10.1, Lt: 9.5 Rt: 11.1, Lt: 9.8	Rt: 19.6, Lt: 19.7 Rt: 14.3, Lt: 15.3 Rt: 9.4, Lt: 10.4	Paraspinal muscle asymmetry >10% was commonly found in men without a history of LBP. This suggests caution in using level- and side-specific paraspinal muscle asymmetry to identify subjects with LBP and spinal pathology

Sions et al. [36]	MRI	13 older adults with chronic LBP, age 60–85 y	L2 L3 L4 L5	Through vertebral body			3.44 ± 0.94 5.07 ± 2.02 8.76 ± 3.02 9.35 ± 1.83	18.76 ± 4.46 17.63 ± 4.00 13.51 ± 2.00 3.61 ± 1.19	

LBP: low back pain, CSA: cross-sectional area, Rt: right side, and Lt: left side.

**Table 2:** CT-evaluated radiographic density of paraspinal muscles and association with LBP.

Research	Participants	Segments measured	Level of measurement	Total paraspinal muscle density (HU)	Partial paraspinal muscle density (HU)^*∗*^	Association with LBP
Keller et al. [15]	31 adults with chronic LBP	T12-L1 L3-L4 L4-L5	Intervertebral disc	Rt: 58.0 (51.7–64.3) Lt: 55.9 (49.5–62.7) Rt: 53.7 (48.1–61.9) Lt: 54.2 (47.5–62.0) Rt: 49.9 (42.6–55.8) Lt: 51.3 (46.1–59.1)	Rt: 63.8 (56.4–71.7) Lt: 59.0 (54.2–66.1) Rt: 57.0 (53.7–64.3) Lt: 59.3 (52.5–63.3) Rt: 51.8 (42.1–58.0) Lt: 52.8 (46.9–62.5)	

Hicks et al. [29]	739 men and 788 women aged 70–79	L4-L5	Intervertebral disc	Men (total): 25.10 ± 9.33 Women (total): 14.98 ± 9.25 No LBP: 19.32 (18.56–20.08) Mild LBP: 17.27 (15.83–18.70) Moderate LBP: 15.99 (14.69–17.28) Severe LBP: 13.42 (11.28–15.56)		Findings suggest a link between trunk muscle composition and history of LBP as well as reduced functional capacity in older adults

Kalichman et al. [5]	187 adults	L3 L4 L5	Intervertebral disc		Values are mean for the three vertebral levels Multifidus male: 64.80 ± 11.05 Multifidus female: 57.12 ± 9.98 Erector spine male: 56.84 ± 11.54 Erector spine female: 52.55 ± 7.41	Paraspinal muscle density decreases with age and increases BMI. It is associated with facet joint osteoarthritis, spondylolisthesis, and disc narrowing but not associated with the occurrence of LBP

Anderson et al. [28]	174 men and women, age 81.9 ± 6.4	L2	Mid-vertebral	Men: 27.2 (9.8) Women: 21.2 (12.1)		

HU: Hounsfield units, LBP: low back pain, Rt: right side, and Lt: left side.

^*∗*^Partial paraspinal muscle density was measured using a 6 mm circle in the center of the most preserved muscle mass.

**Table 3:** Fatty infiltration of paraspinal muscles and association with LBP.

Research	Modality	Participants	Segments measured	Level of measurement	Method	Fatty infiltration		Association with LBP
Fortin et al. [24]	MRI	33 patients diagnosed with posterolateral disc herniation at L4-L5		The center of each intervertebral disc, the center of S1 vertebral body, perpendicular to the muscle mass	The ratio of lean mass CSA to total CSA as an indicator of muscle composition (or fatty infiltration)	Multifidus affected side	Multifidus nonaffected side	Greater fat infiltration on the side and at spinal levels adjacent to the disc herniation. Muscle asymmetry was not correlated with symptom duration
			L3-L4			0.58 ± 0.21	0.61 ± 0.17	
			L4-L5			0.55 ± 0.16	0.57 ± 0.14	
			L5-S1			0.51 ± 0.11	0.53 ± 0.11	
			S1			0.46 ± 0.12	0.49 ± 0.13	
						Erector spine affected side	Erector spine nonaffected side	
			L3-L4			0.58 ± 0.17	0.61 ± 0.14	
			L4-L5			0.47 ± 0.17	0.52 ± 0.12	
			L5-S1			0.30 ± 0.15	0.36 ± 0.15	
			S1			0.29 ± 0.26	0.32 ± 0.17	
					Signal intensity as an indicator for fatty infiltration	Multifidus affected side	Multifidus nonaffected side	
			L3-L4			1959.1 ± 1606.3	1972.6 ± 1610.7	
			L4-L5			2015.3 ± 1811.4	2243.2 ± 1766.0	
			L5-S1			2625.3 ± 2109.2	2476.3 ± 1932.	
			S1			3159.4 ± 2001.5	53029.2 ± 1837.5	
						Erector spine affected side	Erector spine nonaffected	
			L3-L4			1988.0 ± 1593.1	1882.0 ± 1468.3	
			L4-L5			2520.6 ± 2092.6	2338.7 ± 1821.1	
			L5-S1			2876.9 ± 2320.6	2804.5 ± 2200.1	
			S1			3688.4 ± 2137.2	3323.7 ± 1795.4	

Hebert et al. [34]	MRI	401 participants. 40-year-old adults randomly sampled from a Danish population and followed up at 45 and 49 years of age	L4 L5		Using signal intensity to separate muscle from fat. Presented as % of the fat CSA from the total muscle CSA	Out of the four results (level L4, L5: left and right side), only the highest percentage of fat is presented Age 40: 28.8 ± 12.7% Age 45: 28.7 ± 11.9% Age 49: 31.6 ± 13.0%		The relationship between multifidus fat infiltration and LBP/leg pain is inconsistent and may be modified by age

D'hooge et al. [25]	MRI	13 individuals with recurrent nonspecific LBP, and 13 asymptomatic individuals	L3 L4 L4	Superior endplate Superior endplate Inferior end plate Axial images	Muscle-fat-index	Multifidus: LBP: 18.4 ± 6.4 Control: 14.0 ± 2.6 Erector spine: LBP: 23.9 ± 6.1 Control: 20.7 ± 2.5		The increase in fatty infiltration in lean lumbar muscle tissue, in the absence of alterations in muscle size or macroscopic fat deposition after resolution of LBP. It is hypothesized that decreased muscle quality may contribute to the recurrence of LBP

Niemeläinen et al. [35]	MRI	126 asymptomatic men	L3-L4 L4-L5 L5-S1	Not described in the manuscript	The ratio of functional CSA to total CSA as an indicator of muscle composition (or fatty infiltration)	Multifidus: L3-L4; Rt: 82, Lt: 83 L4-L5; Rt: 76, Lt: 77 L5-S1; Rt: 72, Lt: 73 Erector spine: L3-L4; Rt: 84, Lt: 85 L4-L5; Rt: 77, Lt: 79 L5-S1; Rt: 73, Lt: 76		The amount of intramuscular fat significantly increased caudally for both muscles. Paraspinal muscle asymmetry, >10%, was commonly found in men without a history of LBP

Mengiardi et al. [13]	MR spectroscopy	25 patients with chronic LBP and in 25 matched asymptomatic volunteers	L4-5 level		Mean percentage fat content of the muscle	Multifidus: Chronic LBP: 23.6% Control: 14.5% Erector spine: Chronic LBP: 29.3% Control: 26.0%		Significantly higher fat content in the multifidus muscle in patients with chronic LBP than in asymptomatic volunteers

Chan et al. [27]	US, in prone position	12 asymptomatic men; 12 men with LBP	L4 L4	Vertebral lamina	Fat CSA (cm^2^)	Multifidus controls: Lt; 0.56 ± 0.10; Rt; 0.61 ± 0.09 Multifidus LBP: Lt; 1.08 ± 0.23; Rt; 1.13 ± 0.23		Fat area within the multifidus was larger in chronic LBP patients

LBP: low back pain, CSA: cross-sectional area, Rt: right side, and Lt: left side.
